# 1,000 structures and more from the MCSG

**DOI:** 10.1186/1472-6807-11-2

**Published:** 2011-01-10

**Authors:** David Lee, Tjaart AP de Beer, Roman A Laskowski, Janet M Thornton, Christine A Orengo

**Affiliations:** 1 Department of Structural and Molecular Biology, University College London, Darwin Building, Gower Street, London WC1E 6BT, UK; 2 European Bioinformatics Institute, Wellcome Trust Genome Campus, Hinxton, Cambridge CB10 1SD, UK

## Abstract

**Background:**

The Midwest Center for Structural Genomics (MCSG) is one of the large-scale centres of the Protein Structure Initiative (PSI). During the first two phases of the PSI the MCSG has solved over a thousand protein structures. A criticism of structural genomics is that target selection strategies mean that some structures are solved without having a known function and thus are of little biomedical significance. Structures of unknown function have stimulated the development of methods for function prediction from structure.

**Results:**

We show that the MCSG has met the stated goals of the PSI and use online resources and readily available function prediction methods to provide functional annotations for more than 90% of the MCSG structures. The structure-to-function prediction method ProFunc provides likely functions for many of the MCSG structures that cannot be annotated by sequence-based methods.

**Conclusions:**

Although the focus of the PSI was structural coverage, many of the structures solved by the MCSG can also be associated with functional classes and biological roles of possible biomedical value.

## Background

Sequencing of complete genomes has become commonplace in the last decade but traditional methods of protein structure determination cannot keep pace. Structural Genomics (SG) has emerged with the goal of providing a structure for every protein encoded by a genome using a high-throughput combination of experimental structure determination and homology modelling approaches. The Protein Structure Initiative (PSI) is a USA federal government, university, and industry effort that has now completed two phases [1]. The first phase of the PSI (PSI-1), from 2000 to 2005, was dedicated to demonstrating the feasibility of high-throughput structure determination, solving unique protein structures, and developing the methodology and technology for a subsequent production phase. The second phase, PSI-2, focused on implementing the high-throughput structure determination methods developed in PSI-1, as well as homology modelling and addressing bottlenecks like modelling membrane proteins.

There are four large-scale PSI-2 centres and a number of smaller specialist centres. The four large-scale centres are the Joint Center for Structural Genomics (JCSG), the Midwest Center for Structural Genomics (MCSG), the Northeast Structural Genomics (NESG) Consortium, and the New York SGX Research Center (NYSGXRC) for Structural Genomics.

When PSI-1 began at the end of the 20^th ^century there was much optimism that SG could provide structures to cover the whole of protein sequence space and together with improvements in homology modelling technology, it was only a matter of time before a reasonably accurate structure could be predicted for any protein in nature as soon as its sequence was known. It has since become apparent that protein sequence space is much larger than was imagined at the time. Advancements in homology modelling have also not been as great as was hoped. As a consequence SG has failed to deliver the hoped for level of coverage of sequence space and has been left with a collection of structures that much of the time were not targeted on the basis of their biological function. This has led to a criticism of the PSI that many of its structures are of proteins of unknown function and many do not have corresponding publications and therefore give little biological insight. Indeed, the third phase of the PSI is called PSI:Biology, which is intended to reflect a new emphasis on the biological relevance of the work. As PSI-2 draws to a close, we attempt to partially address this problem by exploiting a wide range of bioinformatics tools to provide functional annotations for as many as possible of the protein structures experimentally determined so far by the MCSG.

An early analysis of structures solved worldwide by SG consortia assessed the new coverage of sequence and fold space using the CATH [2] and SCOP [3] domain structure classifications [4]. While SG was judged to be succeeding in structurally characterising new superfamilies, an early observation was that many of the sequences selected as likely to represent new folds were actually found to have existing folds. In 2005 Xie and Bourne adopted a different approach to measuring the impact of SG [5]. They assessed functional coverage of the human genome by existing structures, structural genomics targets, and homology models. Using functional categories in the Enzyme Commission (EC) [6] and Gene Ontology (GO) [7] classifications they showed that, at the time, SG structures provided at least one domain that covered about a third of all the functional classes in the genome, and whole structure coverage for about a quarter of the genome. Even if all the registered SG targets were solved, which was a substantial number of targets even in 2005, then only about two thirds of the functional classes would be covered and there would be whole structure coverage for less than half of the genome. They therefore argued in favour of target selection aimed at functional coverage, especially with a view to understanding human genetic disease.

Also in 2005 Chandonia and Brenner suggested the "Pfam5000" strategy for target selection [8] which involved selecting the 5000 most important Pfam families [9]. This comprehensive collection of protein families is used extensively throughout the biological sciences, often for functional annotation of complete genomes, and it was felt that this strategy would be medically and biologically relevant, of good value, and tractable. Pfam5000 was felt to be better value than the complete solution of several small to moderately sized bacterial proteomes or partial coverage of the human proteome since these would have limited impact on structural knowledge of other proteomes. The JCSG, on the other hand, focused on selecting targets from *Thermotoga maritima *leading to the generation of a three-dimensional reconstruction of the central metabolic network of this bacterium [10]. The JCSG also championed the selection of sequences that were over-represented in the human gut metagenome [11] and this strategy became part of the overall PSI effort.

Chandonia and Brenner went on to analyze the novelty, cost, and impact of SG structures compared to non-SG structural biology (SB) structures [12]. Determination of the first structure in a Pfam family was established as a measure of success. It should be noted, however, that a Pfam family often contains proteins with a range of related functions and finer grained target selection would be necessary to truly achieve complete functional coverage. In 2004 about half of all first structures for Pfam families were from SG rather than SB.

Marsden *et al*. [13] analyzed 203 complete genomes in the Gene3D resource [14] to provide new insights into protein family space. The number of protein families was found to be continually expanding with time but a significant proportion of the proteomes could be assigned to relatively few large, well-characterized domain families while the vast majority of domain families were relatively rare and often species specific. It was suggested that SG could provide structures for fewer than a thousand Pfam families to achieve reasonable structural coverage of genomes. Within these large families it was suggested that multiple structures could be determined to reveal more about the evolution of the family and enable greater understanding of how function evolves. Similarly, the selection of targets from within large and diverse CATH superfamilies was proposed to maximize modelling leverage [15]. Since many of the targets previously selected as likely to have a novel fold proved to have a known fold this could be seen as a more rational use of resources.

There was a coordinated strategy of target selection by the four large-scale centres during PSI-2, described by Dessailly *et al*. [15], involving four main approaches: targeting representatives from large, structurally uncharacterized protein domain superfamilies; targeting structurally uncharacterized subfamilies in very large and diverse superfamilies with incomplete structural coverage; community nominated targets; and biomedically important targets. The first two approaches were primarily aimed at increasing the structural coverage of protein sequence space and many targets were selected regardless of their function. Metrics have been calculated in this and several other publications [16-18] to show that this strategy has had the result that PSI's per structure contribution to novel structural leverage has been much higher than that for SB, as would be expected since SB is not aiming to increase sequence coverage but rather provide biological insights.

The Structural Biology Knowledgebase [19,20] offers an easy way of keeping abreast of developments by the PSI and by SG and SB in general. It is a continually updated portal to research data and other resources from the PSI. Metrics are regularly updated giving a summary of the total number of structures, the number of distinct and novel structures determined by the PSI, and the modelling leverage that PSI structures provide. Models are made available through the Protein Model Portal [21,22]. The JCSG has made a notable effort towards the annotation of PSI structures with The Open Protein Structure Annotation Network (TOPSAN), a wiki-based portal for the dissemination of information for the broad biomedical community [23].

The increased appearance of structures of proteins of unknown function in the PDB due to SG has stimulated the development of computational methods of function prediction from structure. ProFunc, developed for the MCSG, combines a number of sequence-based and structure-based methods to gain clues about the function of a protein [24]. The MCSG PSI-1 structures were used to test and develop ProFunc [25]. When a sequence match is weak and/or multiple functions are suggested, the structure-based methods within ProFunc can help select from the options that are presented and increase confidence in a particular putative function. Another recent and notable method that has been used for function prediction from PSI structures is FLORA [26]. This uses structural motifs associated with different functional sub-groups within functionally diverse CATH domain superfamilies.

The MCSG selected targets from a broad range of pathogenic bacteria according to selection criteria that have evolved throughout the first two stages of the PSI. Here we attempt to extend the functional annotations associated with MCSG structures by employing a diverse set of bioinformatics tools and resources. We also analyse the structural novelty of protein structures solved by the MCSG. We use sequence-based methods to assess the proportion of MCSG proteins that have a known function, a putative function, a possible function, and no known function. This is followed up with ProFunc analysis to support some of the possible functional annotations and in some cases to suggest possible functions for the unknowns.

## Methods

A list of all protein structures released by the PDB [27], their release date, and the source SG centre where applicable was downloaded from the PDB on the 3^rd ^March 2010. In all subsequent analyses only data that were available on this date are used. At the time 1,165 MCSG protein structures for 1,118 targets had been released by the PDB.

### Annotations

The source of most of the annotations used in this work is Gene3D 9.1.0 [14]. This database contains nearly 10 million protein sequences including all UniProt sequences [28] and most complete genomes. Annotations are imported from multiple sources including EC numbers [6] from UniProt, GO terms [7], KEGG genes [29] and protein family assignments from Pfam [9], TIGRFAMs [30], SMART [31], and PANTHER [32]. KEGG currently has 357 reference pathway maps. These are represented in a general way to be applicable to all organisms and thus are useful in the analysis of SG structural coverage. Each node in a reference pathway is represented by one or more manually curated KEGG orthologs. Where there are multiple orthologs these may represent different subunits or different versions of the same enzyme. Also a node may belong to more than one pathway when different pathways interact. KEGG genes and orthologs are mapped to genomes in Gene3D and PDB chains are mapped to UniProt entries using http://www.ebi.ac.uk/thornton-srv/databases/pdbsum/data/pdb_chain_sp_ec the data for which comes from the PDBe database [33]. In addition to collecting these annotations from Gene3D for MCSG structures, annotations are considered for inheritance by sequence-sequence comparison, sequence-profile comparison, and profile-profile comparison, such that increasingly remote sequence similarities are considered.

Not all assignments to a protein family lead to a functional annotation. Some assignments are filtered out as being uninformative *e.g*. assignments to a Pfam family described as a DUF (domain of unknown function). The sequence of each MCSG structure is compared to all sequences in Gene3D using BLAST (sequence-sequence comparison) and five iterations of PSI-BLAST (sequence-profile comparison), both with an E-value cut-off of 0.01 [34]. EC numbers and molecular function GO terms associated with matching sequences are considered for inheritance. Both of these types of annotation are hierarchic. EC numbers all have four levels, the first level refers to the enzyme class ('1,' for example, refers to oxidoreductases), the second level refers to the type of bond or group that is acted on ('4,' for example, denotes a peptide bond), and the next two levels give progressively more specific details of the catalyzed reaction and its substrates. GO molecular function terms are organized as a directed acyclic graph where there may be many nested levels with each child having an 'is a' relationship to its parent. Annotations associated with a sequence or sequence match are divided into deep annotations comprising level 3 or 4 EC numbers and GO terms at level 6 or above, and the remainder which are generally less specific annotations. Hidden Markov models (HMMs) are built using the sequence of each MCSG structure as a seed for SAM-T [35] and then each of these HMMs is compared to all Pfam 24.0 HMMs (profile-profile comparison) using PRC [36].

Database annotations, inherited annotations, and annotations from the headers of the PDB files are presented together in a table at http://www.biochem.ucl.ac.uk/cgi-bin/dlee/MCSG_annotations used by the authors to manually assign an annotation status to each MCSG structure. Annotations are organized from left to right to reflect decreasing levels of both depth and reliability. Four levels of status are assigned; known, putative, possible, and unknown. GO provides evidence codes for functional annotations where the most reliable codes are the experimental evidence and author statement codes. 62% of MCSG solved targets have a molecular function GO term associated with them but only 2% have such a high quality functional annotation, the remainder have annotations that are electronically inferred. There are currently very few other annotation resources where evidence codes are available and sequence coverage is low. We consider possession of one of the most reliable GO evidence codes as too strict a criterion to define the 'known' status. Rather, possession of a deep database annotation and preferably agreement with at least one set of protein families is generally taken as evidence that the function is regarded as being known. Note that a GO term level of 6 or higher is only a rough guide to the depth of an annotation and the association of a term such as GO:0005506 for iron ion binding (level 7) is not considered to be sufficient in itself for a 'known' status. This is one reason why manual assessment of status is so important. The 'putative' status is generally assigned where there is no deep database annotation but there is a protein family assignment and the family description includes specific molecular functions. The 'possible' status is generally assigned where only inherited annotations are available but without the added confidence provided by assignment to a family in a curated resource. There may be a choice between a variety of deep annotations or perhaps only a very general, non-specific annotation is available presenting a broad range of possible deep annotations. The 'unknown' status is applied where sequence methods are unable to provide any clue to the function of the protein. ProFunc analysis (see below) is linked to the online table for structures assigned a 'possible' or 'unknown' status.

GO slims are cut-down versions of the GO ontologies containing a subset of the terms in the whole GO. The GO provides a generic GO slim that gives a broad overview of all function categories without the detail of the specific fine grained terms. This is useful for visualization of the broad functional coverage of MCSG structures and for comparison to the functional coverage of the PDB as a whole. A sequence unique subset that approximately represents all PDB protein entries of known function is generated by selecting representatives annotated in Gene3D with a molecular function GO term at level six or higher. GO terms associated with these representatives are then mapped to the generic GO slim using the GO Slimmer tool at http://amigo.geneontology.org/cgi-bin/amigo/slimmer. Similarly the UniProt entries for all MCSG solved targets with known function manually assigned as above are mapped to the generic GO slim.

### ProFunc analysis

The ProFunc web server at http://www.ebi.ac.uk/profunc[24] employs a number of complementary function-prediction methods, focusing, in particular, on methods based on 3D structural information. This accepts a PDB file and runs a number of sequence- and structure-based analyses on it, listing any hits it finds to existing sequences and structures in descending order of significance. These can help researchers identify any strong similarities which may be indicative of the protein's function.

Of ProFunc's structure-based methods, the two that have been shown most accurately to suggest function [25] are the SSM (Secondary Structure Matching) fold-matching algorithm [37] and the "reverse template" search [38]. Both these methods have a similar success rate and are able to find distant homologues when simple sequence-based searches fail. The majority of their hits overlap, but occasionally one method picks up a match that the other fails to identify. The advantage of the reverse template method over the fold-match is that its matches are local and the top-scoring hits tend to pick up sites of functional relevance.

To demonstrate the added value of 3D structure for proteins of uncertain or unknown function, we run ProFunc on the full data set of 1,165 MCSG structures. ProFunc results for targets assigned a 'possible' or 'unknown' status following sequence analysis are linked to the online table. ProFunc results for targets of 'unknown' status are manually assessed to determine the improvement in functional coverage afforded by ProFunc for MCSG solved targets.

To further illustrate the value of ProFunc analysis we then focus on MCSG structures where no significant match to a known 3D structure in the PDB can be found from a search based on sequence alone, but is detectable using the structure-based reverse template method. We investigate which parts of the matched structures the method identifies as the most significantly similar.

### Reverse templates

The reverse template method takes the query protein structure and breaks it up into a large set (typically several hundred) of 3-residue templates. Each template consists of three neighbouring residues chosen such that the closest atom-atom distance between any two of the residues is no larger than 5.0Å. Templates containing more than one hydrophobic residue are rejected in order to bias the templates towards surface residues. A scan of each template against a representative set of the structures in the PDB is then made using the Jess algorithm [39] to identify structures containing similar constellations of the three residues. The representative set is a non-redundant list of protein chains in the PDB downloaded weekly from the Pisces ftp server [40]. Hits are scored and assigned E-values as described in Laskowski *et al*. [38], the scoring being based on the local similarity of the equivalent regions when the structures are superposed on the matched residues.

The aim of the method is to pick up local structural similarity between two proteins and, in particular, to identify regions that have been conserved by evolution and hence are more likely to be functionally important. Thus, even though the sequences of two homologous proteins might have diverged considerably over evolutionary time, it is possible that, to maintain their function, the proteins' active sites have undergone less change. The detection of such local conservation can provide strong support for the proteins having retained the same, or a similar, function. 3 residues and a 5.0Å cut-off were found through empirical testing to give a good balance between the duration and success of a search (unpublished data).

### Identification of functionally important regions

To demonstrate this tendency to pick up functionally important regions, we select a subset of the MCSG data where a simple FASTA [41] search against the sequences in the PDB fails to return a significant result. We use an E-value cut-off of 0.01, which gives us 336 structures whose best match is at or below this threshold.

We then consider the top four hits returned by the reverse template search for each of these 336 structures to see if the template residues have any association with the protein's function. We use the functional annotations given in PDBsum [42], namely: residues belonging to a PROSITE [43] pattern; catalytic residues, as determined from the Catalytic Site Atlas (CSA) [44]; residues defined in the SITE records of the original PDB file; or residues in contact with a ligand, metal or DNA/RNA in the structure.

However, as the search data set used by the reverse template method is a representative one, it is possible that the PDB entry matched by the search has no functional annotation, yet a closely related PDB entry, excluded from the data set, does have some annotation. There were nearly 28,000 protein chains in the representative data set, compared with over 64,000 structures in the PDB. Thus, to pick up such 'lost' annotations, we use the Sequence Annotated by Structure (SAS) server [45] to find additional functional annotations for each of the matched structures. SAS identifies closely related sequences from the PDB using FASTA. Functional annotations are then imported from the resultant alignment(s) where the sequence identity is at least 30%, the alignment overlap at least 80 residues, and the FASTA E-value < 0.001.

### Functional novelty

The release dates of all PDB entries that are associated with a GO term [7], are assigned to a Pfam family [9], or map to KEGG orthologs [29] are compared to find the earliest example released by the PDB for each annotation. Each structure that represents a first example is then categorized as being solved by a PSI centre or as 'non-PSI' in order to rate the performance of PSI compared to the combined effort of all other laboratories, and to compare the performance of individual PSI centres. The dates that the first structures were solved are also divided into the two PSI periods PSI-1 (before 1^st ^July 2005) and PSI-2. A measure of novelty per PDB release is calculated and for comparison the novelty per non-redundant structure at 95% sequence identity following clustering using cd-hit is also given[46].

### Structural novelty

The release dates of all PDB entries containing a domain assigned to a superfamily in CATH v3.3 are compared to find the earliest entry in that superfamily (CATH number) and fold (CAT number). Structural novelty within a superfamily is determined by calculating a normalized RMSD (normRMSD). A domain with a normRMSD of 5Å or more from all CATH domains in structures previously released by the PDB is defined as structurally novel and belongs to a new structural sub-group (SSG). Both MCSG domains assigned to CATH and unassigned MCSG PDB chains are compared to a representative library of CATH domains (S35 reps) using CATHEDRAL [47] as a fast filter for significant structural similarity. The best matches by CATHEDRAL are then structurally aligned and superposed using the more computationally expensive and accurate method SSAP [48] and the RMSD is calculated. The normRMSD is then calculated as follows:

where *RMSD *is the root mean square deviation of the superposition, max (*L*_1_, *L*_2_) is the length in residues of the longest domain in the superposition, and *N_mat _*is the number of aligned residue pairs (Kolodny *et al*. 2005) [49].

### Novel modelling leverage

Novel modelling leverage is calculated according to the method of Nair *et al*. [18]. The sequences of all PDB protein chains, non-redundant at 100% sequence identity, are compared to UniRef100 sequences downloaded on the 3^rd ^of March 2010 using PSI-BLAST with three iterations and an E-value cut-off of 1e-10. Novel modelling leverage in residues is determined for each PDB entry (including redundant entries) on the date of its release by the PDB. The novel modelling leverage of the four large-scale PSI centres is compared to each other and to the leverage of the combined non-PSI laboratories.

### Human non-synonymous single nucleotide polymorphisms

The MCSG focused on solving structures of proteins from pathogenic bacteria and has only solved structures for two human proteins. However, a number of MCSG structures may be used as templates for comparative modelling of human proteins. All such structures identified in the modelling leverage analysis described above are used to analyze human non-synonymous nucleotide polymorphisms (nsSNPs). MCSG structures are included regardless of whether a closer template is available from another laboratory.

For each human modelling target identified above, variants are retrieved from UniProt, Ensembl and OMIM and filtered for uniqueness. UniProt variants and Ensembl gene IDs are listed for each protein in the UniProt text file available from UniProt. The Ensembl API is used to query their MySQL database and retrieve all Ensembl variants. OMIM variants are linked to UniProt IDs through the OMIM Missense server at http://www.bioinf.org.uk/omim/. Each variant is inspected to see if it falls within a region having a template match and is thus modellable.

## Results and Discussion

### Annotations

**Figure 1:**
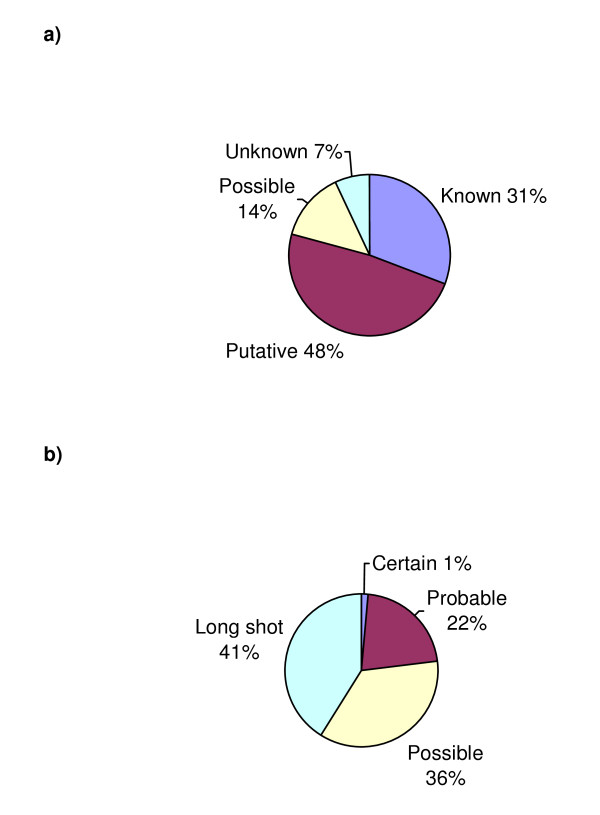
**Functional annotation coverage of MCSG structures**. **a) **Pie chart showing the proportion of MCSG targets manually assigned as having a known, putative, possible, or an unknown function. **b) **Pie chart showing the likelihood of the best scoring ProFunc template-search prediction being correct for the targets that are of unknown function following annotation by sequence methods.

The functional annotation status of the MCSG solved targets is as follows: 31% known; 48% putative; 14% possible; and 7% unknown (See Figure 1 a). ProFunc analysis of the unknowns is summarised in Figure 1 b. The majority of structures are therefore of direct relevance to biological investigations of protein function as well as fulfilling some of the goals of SG in expanding structural coverage of fold and function space as shown below.

**Figure 2:**
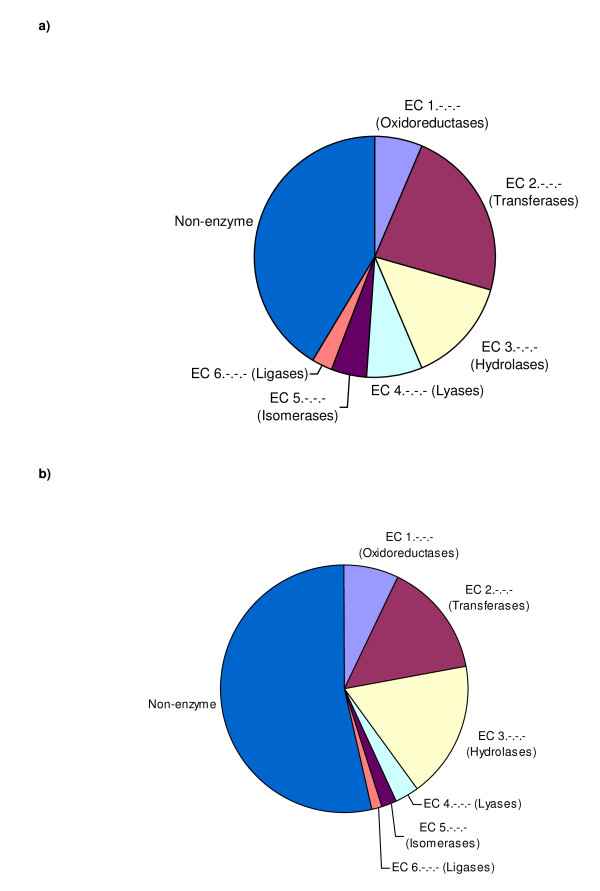
**EC classes of MCSG structures compared to the PDB as a whole**. **a) **Pie chart showing the distribution of EC classes for the MCSG structures that have a known function. **b) **Pie chart showing the distribution of EC classes for all PDB entries taken from the Enzyme Structures Database at the EBI http://www.ebi.ac.uk/thornton-srv/databases/enzymes/.

**Figure 3:**
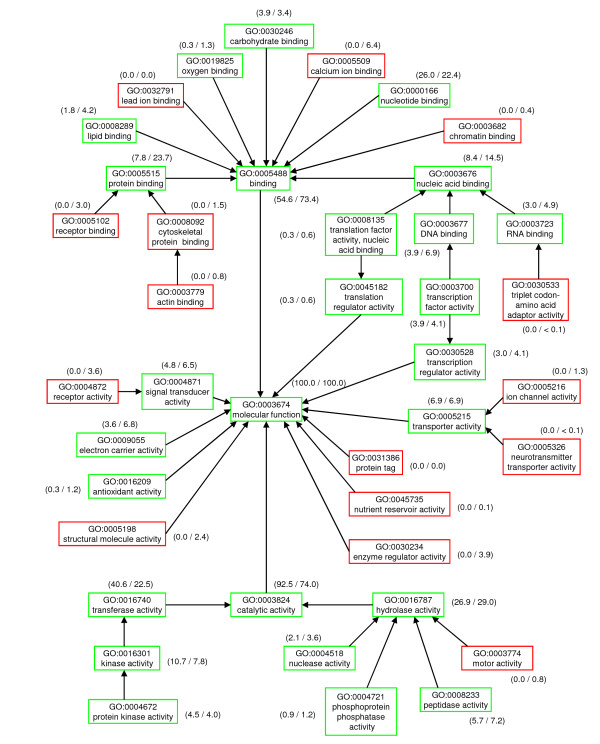
**Distribution of molecular function GO terms associated with MCSG structures**. The molecular function ontology terms of the generic GO slim give a broad overview of all protein molecular function categories. Arrows indicate 'is a' relationships. MCSG structures with a known function cover terms in boxes with a green border while terms in boxes with a red border are not covered by MCSG structures. Numbers in parentheses outside of each box show the proportion (%) of MCSG targets with a solved structure and known function that are associated with each term compared to the proportion of all sequence unique PDB structures of known function (MCSG%/PDB%). Note that a structure may be associated with multiple GO terms and many sub-categories of GO terms are not represented in the generic GO slim so the illustrated percentages do not necessarily add up to the totals for their illustrated parent categories.

Figure 2 suggests that the MCSG has solved proportionately more enzyme structures compared to the average contributor to the PDB. A higher proportion of those enzymes are transferases, lyases, isomerases and ligases at the expense of oxidoreductases and hydrolases. The molecular function ontology terms of the generic GO slim in Figure 3 give a broad overview of all protein functional classes including the non-enzyme functional classes that, by definition, are not covered by the EC classification. MCSG structures show broad functional diversity by covering more than half of the generic GO slim terms and nearly a half of its leaf node terms, these being the more functionally specific levels. Use of the generic GO slim is also a test for bias in the functional classes covered by the MCSG structures. Compared to the PDB as a whole, a higher proportion of MCSG structures are associated with the catalytic activity category which is in agreement with the EC analysis. This is at the expense of a generally lower level of association with the other major functional classes, especially binding, structural molecule activity, enzyme regulator activity, and electron carrier activity.

The apparent discrepancy between the results obtained by looking at EC numbers in Figure 2 and GO terms in Figure 3 needs explaining. EC numbers are very high quality annotations but coverage of the PDB is less than that of GO and some catalytic activity is not represented by EC numbers. Within the GO catalytic activity category an over-representation of transferase activity and an under-representation of hydrolase activity is seen which does broadly agree with the EC analysis. Within the GO binding category MCSG structures also show particularly significant under-representation of protein binding and calcium binding while nucleotide binding is actually a little over-represented.

### ProFunc analysis

ProFunc's template searches are rated as certain, probable, possible, or long shots to indicate the likelihood that a prediction is correct. ProFunc analysis is linked to all structures assigned a "possible" or "unknown" status in the online table at http://www.biochem.ucl.ac.uk/cgi-bin/dlee/MCSG_annotations ProFunc suggests functions for all of the 78 MCSG targets that are of unknown function following sequence analysis. The overall likelihood of the best scoring ProFunc prediction being correct is shown in Figure 1 b. One target has a prediction rated as being certain while another 17/78 (22%) targets have predictions rated as being probable. These predictions are all made by the reverse templates method while two of the probable predictions are also made by ligand-binding templates. ProFunc makes a valuable contribution to functional annotation coverage of MCSG structures.

**Figure 4:**
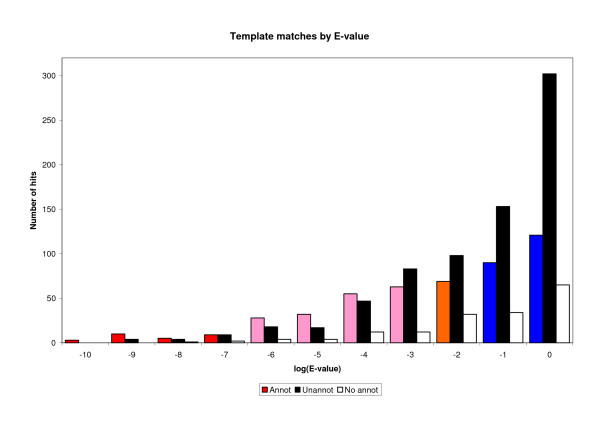
**Identification of functionally annotated residues as a function of the reverse template E-value**. Histogram showing the relationship between the E-value of the reverse template hit and how often it matches a functionally annotated residue in the matched structure. The bars show: the number of times one of the matched residues was functionally annotated (coloured), the number of times none were annotated (black), and the number of time the matched structure had no annotation whatsoever (white). The colours correspond to the 'confidence class' of the reverse template hit, which is determined by its E-value: red = certain match (E < 10^-6^); pink = probable match (10^-6 ^< E < 10^-2^); orange = possible match (10^-2 ^< E < 10^-1^); and blue = long shot (10^-1 ^< E < 10).

Figure 4 demonstrates that the reverse template matches tend to hit functionally important residues and so predict the likely location of the protein's functional site as well as its overall function. Using a subset of 336 MCSG structures where a FASTA search against the PDB fails to return a significant match, the matches returned by the reverse templates were analysed. The residues matched by the top 4 template hits were checked for functional annotation. Figure 4 show that at very low E-values the annotated residues that are hit (coloured bars) tend to outnumber the residues with no annotation (black bars). As the E-value increases so the proportion of hits to non-functional matches rises. A fraction of the cases (17%) hit protein structures having no annotation at all, so these are shown separately (white bars). Thus the better the reverse template match, the more likely it is to hit a functionally important part of the protein. This is what one would expect if the functional parts of distantly related proteins change less than the rest of the protein due to evolutionary pressure to preserve the same, or similar, function.

### ProFunc example

**Figure 5:**
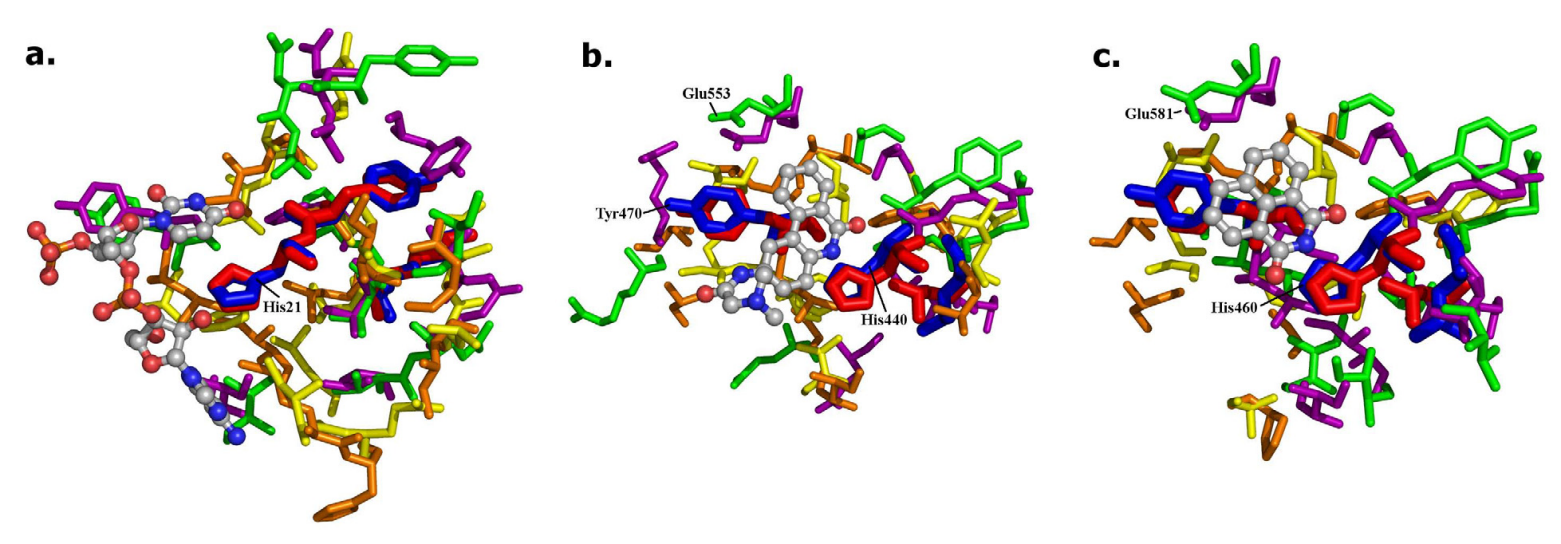
**Prediction of function from structure using ProFunc**. Three reverse template matches for PDB entry 2aua, a protein of unknown function from Bacillus cereus. The matches are to the catalytic domains of three toxins: a) diphtheria toxin from *Corynebacterium diphtheriae *(PDB code 1f0l), b) exotoxin A from Pseudomonas aeruginosa (PDB code 1 × k9) and c) cholix toxin from Vibrio cholera (PDB entry 3ess). In each case, the template residues from the 2aua query structure are shown in thick, red sticks while the corresponding residues in the target structure are shown as thick, blue sticks. Neighbouring identical residues, in equivalent 3D positions, are shown in purple for 2aua and green for the target, while similar residues are shown in orange for 2aua and yellow for the target. The inhibitor molecules bound in the target structures are shown in ball-and-stick representation and are: a) adenylyl-3'-5'-phospho-uridine-3'-monophosphate, b) N-(6-oxo-5,6-dihydro-phenanthridin-2-yl)-N,N-dimethylacetamide and c) 1,8-naphthalimide. Catalytic residues are labelled using the residue numbering of the corresponding PDB entries.

An example of a reverse template match is PDB entry 2aua which is the structure of BC2332, a protein from *Bacillus cereus *that is classified as 'unknown' by the sequence analysis described above. The reverse template search finds one 'certain' match, one 'probable' match and seven 'possible' matches. Figure 5 shows the template residues and surroundings for three of these matches, all being to the catalytic domains of bacterial toxins. The first is diphtheria toxin (a 'certain' match), PDB code 1f0l [50], and the other two are 'possible' matches: one to exotoxin A from *Pseudomonas aeruginosa*, PDB code 1 × k9 [51] and the second to cholix toxin from *Vibrio cholera*, PDB entry 3ess [52]. Each of the templates includes one or more of the known catalytic residues, and the local region returned by the template match corresponds to the matched protein's known substrate binding site. The catalytic domains correspond to EC 2.4.2.36, which contains the NAD^+^-diphthamide ADP-ribosyltransferases. All have the same alpha-beta fold, CATH 3.90.175.10, corresponding to domain 1 of diphtheria toxin. These matches strongly suggest that the BC2332 protein has a very similar function to these toxins. *Bacillus cereus *produces a number of known enterotoxins that are responsible for food poisoning [53]. Possibly, this represents another.

A second ProFunc example is presented [Additional file 1] to show how ProFunc analysis can add weight and specific detail to a possible annotation suggested by sequence analysis.

### Functional novelty

So far 5,699 out of 11,912 Pfams, 2,938 out of 9,929 GO molecular function terms and 2,049 out of 7,080 terms at level 6 or higher have at least one representative with a solved structure. Note, however, that because it is generally only small Pfam families that lack a structural representative, 88% of unique Pfam assignments in complete genomes in Gene3D belong to a Pfam family with a structural representative. Table 1 gives an indication of the value of PDB releases in terms of functional novelty. From an SG point of view the number of novel structures is given as a percentage of the total number of structures. SB laboratories often solve multiple structures of the same protein with different ligands bound, at a different pH, or with single amino acid substitutions *etc*. These investigations have different goals from those of SG but here the intention is to assess the performance of PSI centres in terms of SG goals. For an SB point of view the number of novel structures is given for comparison as a percentage of the number of non-redundant structures at 95% sequence identity. Although PSI centres did not specifically target structurally uncharacterized GO terms their overall performance is at least as good as that of all non-PSI laboratories combined in terms of SG goals.

**Table 1:** Functional novelty of structures.

Annotations and period	Source	First structures	Structures		% first	
			
			total	nr	total	nr
GO terms during PSI	MCSG	45	1165	1110	3.86	4.05
	
	JCSG	33	987	938	3.34	3.52
	
	NESG	23	808	720	2.85	3.19
	
	NYSGXRC	35	883	789	3.96	4.44
	
	PSI	153	4101	3711	3.73	4.12
	
	Non-PSI	1677	49373	22192	3.40	7.56

GO terms during PSI-1	MCSG	25	274	265	9.12	9.43
	
	JCSG	18	181	169	9.94	10.65
	
	NESG	15	178	170	8.43	8.82
	
	NYSGXRC	19	187	169	10.16	11.24
	
	PSI	82	872	811	9.40	10.11
	
	Non-PSI	1189	20803	10319	5.72	11.52

GO terms during PSI-2	MCSG	20	891	852	2.24	2.35
	
	JCSG	15	806	772	1.86	1.94
	
	NESG	8	630	558	1.27	1.43
	
	NYSGXRC	16	696	624	2.30	2.56
	
	PSI	71	3229	2964	2.20	2.40
	
	Non-PSI	488	28570	14975	1.57	3.26

Pfam families during PSI	MCSG	241	1165	1110	20.69	21.71
	
	JCSG	133	987	938	13.48	14.18
	
	NESG	197	808	720	24.38	27.36
	
	NYSGXRC	131	883	789	14.84	16.60
	
	PSI	726	4101	3711	17.70	19.56
	
	Non-PSI	3028	49373	22192	6.13	13.64

Pfam families during PSI-1	MCSG	108	274	265	39.42	40.75
	
	JCSG	36	181	169	19.89	21.30
	
	NESG	69	178	170	38.76	40.59
	
	NYSGXRC	35	187	169	18.72	20.71
	
	PSI	260	872	811	29.82	32.06
	
	Non-PSI	2069	20803	10319	9.95	20.05

Pfam families during PSI-2	MCSG	133	891	852	14.93	15.61
	
	JCSG	97	806	772	12.03	12.56
	
	NESG	128	630	558	20.32	22.94
	
	NYSGXRC	96	696	624	13.79	15.38
	
	PSI	466	3229	2964	14.43	15.72
	
	Non-PSI	959	28570	14975	3.36	6.40

Numbers of first structures and their percentage of total structures and non-redundant (nr) structures at 95% sequence identity for molecular function GO terms and Pfam families during PSI, PSI-1, and PSI-2.

Pfam families, on the other hand, were targeted by PSI centres and the proportion of first structures solved out of all structures solved is significantly higher for PSI than for non-PSI laboratories as would be expected. As would also be expected, the proportion of first structures for Pfams are considerably higher than those for GO terms since GO terms are a much more general functional category compared to the evolutionarily related members of Pfams. The further back in time that we go the fewer is the number of GO terms and Pfams that were structurally characterized and thus there was a higher probability of solving the first structure for a term or family. As structural coverage becomes more complete the probability of solving the first structure for a term or family diminishes. This is one likely reason that the rate of solving first structures during PSI-1 is higher than that during PSI-2 for both PSI and non-PSI laboratories. Another possible explanation is that the easier functions have been solved and the remaining functions are more difficult to obtain a structure for. Perhaps we are seeing a combination of both factors. By virtue of solving more structures than any other individual PSI centre the MCSG also solved the highest number of first structures for both GO terms and Pfams. In both cases the proportion of first structures is close to the average for all PSI centres although they excelled at solving the first structures for Pfams during PSI-1.

39/45 of the MCSG first structures for GO molecular function terms correspond to a level 4 EC number and 3 of the remaining 6 also have catalytic activity *e.g*. GO:0015424 amino acid-transporting ATPase activity, again showing a bias towards enzymes. The EC and GO annotation systems have different coverage from each other. Analysis of EC numbers in Gene3D shows that the MCSG solved the first structure for 32 rather than 39 level 4 EC numbers. 5 of these EC numbers were not revealed by the GO analysis so in total the MCSG solved the first structure for 44 level 4 EC numbers. The novelty of these structures may also be considered in terms of providing structural coverage for metabolic pathways.

### Pathways

There are currently 357 KEGG reference pathways. These pathways have nodes, most but not all of which correspond to EC numbers. KEGG orthologs representing evolutionarily related groups of proteins map to many but not all of these nodes and many nodes have multiple orthologs. Currently, 7,035 orthologs are assigned to the reference pathways and more are likely to be added in the future. 1,849 out of a total of 4,977 currently possible level 4 EC numbers map through KEGG orthologs to KEGG reference pathway nodes. 1,147/1,849 (62%) have at least one structural representative compared to 1,670/4,977 (34%) for the EC classification as a whole. 9/357 (3%) pathways have complete structural coverage of their orthologs but these are all small pathways, the largest having only 8 orthologs belonging to 6 nodes, ko00072 synthesis and degradation of ketone bodies. This pathway is also the largest pathway composed entirely of nodes corresponding to level 4 EC numbers with complete structural coverage.

The 44 nodes with EC numbers that the MCSG solved the first structure for belong to 44 KEGG reference pathways but this number is a coincidence, with some of the nodes appearing in multiple pathways while multiple nodes also appear in single pathways. The largest number of nodes within a single KEGG reference pathway to gain a first structure from the MCSG is 6 for pathway ko00330, arginine and proline metabolism, but this is a large pathway with more than 100 nodes. PSI centres did not select targets with the aim of achieving complete coverage of metabolic pathways but this is a potential goal for the future.

Overall the MCSG solved the first structure for 73 orthologs in 59 pathways. 43 of these orthologs belong to multiple pathways and thus join pathways together. Membership of multiple pathways is, however, not rare with about a half of all KEGG orthologs (3,514/7,053) belonging to more than one reference pathway.

### Structural novelty

Of the 1,165 MCSG PDB entries analyzed in this work only 513 are so far classified in CATH v3.3 and so the analysis of structural novelty is currently incomplete. Also, since a special effort was made by the curators of CATH to update the classification of MCSG structures as a priority over other structures, MCSG structural novelty cannot be compared to that of other PSI centres or SB in general. MCSG structural novelty is shown in Table 2.

**Table 2:** Structural novelty of MCSG structures.

Level of structural novelty	Number of domains (cumulative total)
**Fold**	54

**Superfamily**	66 (120)

**Structural sub-group (SSG)**	52 (172)

Number of novel CATH structural domains solved by the MCSG. Three levels of novelty are defined: a domain with a novel fold is the first example released by the PDB of a fold in CATH (a new CAT number); a domain belonging to a novel superfamily is the first example released by the PDB of a superfamily in CATH (a new CATH number); a domain belonging to a novel structural sub-group (SSG) has a normalized RMSD of at least 5Å from all previously existing domains in CATH.

By definition all new folds are also new superfamilies and all new superfamilies are also new structural subgroups (SSGs). However, in this analysis not all new SSGs are classified in CATH or necessarily belong to existing superfamilies or folds. Of the 52 domains that belong to new SSGs but are not classified in CATH as new folds or superfamilies, 17 are found to be classified into pre-existing CATH superfamilies while 35 are not classified in CATH v3.3. 11 of the 35 not classified achieve a score >70 from the structural comparison program SSAP following the CATHEDRAL scan suggesting that they probably do belong to pre-existing superfamilies. The remaining 24 domains could potentially represent new superfamilies or folds.

### Novel modelling leverage

The average novel modelling leverage in residues per structure is considerably higher during PSI-1 compared to PSI-2 for both PSI and non-PSI (See Table 3). This is despite total modelling leverage per structure actually being higher for both PSI and non-PSI during PSI-2 compared to during PSI-1 (results not shown). The reason is that as the cumulative total and diversity of solved structures increases and targets with good modelling leverage are preferentially selected in the early stages of PSI, any new structures that are solved have less chance of providing novel modelling leverage for a fixed set of protein sequences. During both PSI-1 and PSI-2, PSI substantially outperformed non-PSI in terms of novel modelling leverage per structure as would be expected since non-PSI often target multiple forms of a given target protein to better characterise the biology. With the increased output of PSI during PSI-2, the contribution of PSI to total worldwide novel modelling leverage more than doubled from 7.6% during PSI-1 to 16.3% during PSI-2. During both PSI-1 and PSI-2, the MCSG substantially outperformed the other three large-scale centres in terms of both total novel modelling leverage and average novel modelling leverage per structure (see Table 3).

**Table 3:** Novel modelling leverage.

Period	Source	Novel modelling leverage (residues)	Total structures	Average novel modelling leverage per structure (residues)
PSI	MCSG	59597162	1165	51156
	
	JCSG	33162765	987	33600
	
	NESG	26001821	808	32180
	
	NYSGXRC	31228566	883	35366
	
	PSI	155344631	4101	37880
	
	Non-PSI	1314954328	49373	26633

PSI-1	MCSG	30379825	274	110875
	
	JCSG	14292241	181	78963
	
	NESG	11647910	178	65438
	
	NYSGXRC	16657925	187	89080
	
	PSI	74059521	872	84931
	
	Non-PSI	897493690	20803	43143

PSI-2	MCSG	29217337	891	32792
	
	JCSG	18870524	806	23413
	
	NESG	14353911	630	22784
	
	NYSGXRC	14570641	696	20935
	
	PSI	81285110	3229	25173
	
	Non-PSI	417460638	28570	14612

Novel modelling leverage for residues in UniRef100 during PSI, PSI-1, and PSI-2.

### Human non-synonymous single nucleotide polymorphisms

In the modelling leverage analysis 152 MCSG structures are identified as templates for 867 human proteins in UniProt. In this analysis of modellable nsSNPs all modelling leverage of MCSG structures is considered, not just novel leverage, and the MCSG structure is not necessarily the closest available template. Within these human protein sequences 8,982 unique Ensembl nsSNPs, 1,580 unique UniProt nsSNPs, and 191 unique OMIM nsSNPs are identified. 2,252 of these nsSNPs are within a modellable region when using an MCSG structure as a template.

A good example of an MCSG structure helping to explain human disease is illustrated by PDB entry 2hma. Human mitochondrial tRNA-specific 2-thiouridylase 1 (UniProt ID O75648) matches 2hma with 42% sequence identity in a BLAST alignment. The enzyme has been implicated in aggravating mitochondrial 12S ribosomal RNA aminoglycoside-induced and non-syndromic deafness. It catalyzes the 2-thiolation of uridine at the wobble position (U34) of mitochondrial tRNA(Lys), tRNA(Glu) and tRNA(Gln). A few natural variants have been identified and one (Ala10Ser) has been linked to a decrease in enzymatic activity.

**Figure 6:**
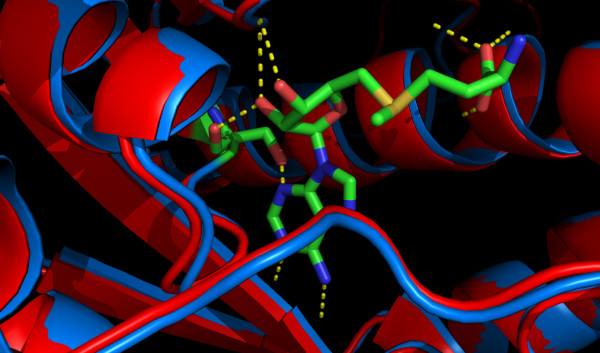
**Explaining the effect of an nsSNP using a homology model based on a MCSG structure**. The interaction between S-adenosyl methionine (SAM) and mitochondrial tRNA-specific 2-thiouridylase 1. The Ala10Ser variant probably introduces a hydrogen bond between SAM and the enzyme that increases binding affinity and thus slows down SAM release hence reducing activity. The wild type model is shown in red and the Ala10Ser variant is shown in blue. The variant residue and SAM are coloured according to their atom types and potential hydrogen bonds are shown in yellow.

To attempt to explain this, a homology model is built using 2hma as a template which includes the substrate, S-adenosyl methionine (SAM). When inspecting the model, it is clear that Ala 10 is part of the SAM binding pocket of the enzyme (See Figure 6). In 2hma the adenosine group fits neatly into this pocket where Ala 10 is located. In the Ala10Ser mutation, an additional hydrogen bond between the protein and SAM is probably introduced. This increases the binding affinity between SAM and the protein and thus slows down the release of SAM once the sulphur has been transferred. This agrees with observations made by Guan *et al*. [54] that the Ala10Ser mutation reduces activity.

## Conclusions

The MCSG has performed well during the first two phases of the PSI in terms of the goals that were established during this period. Whilst there has been concern that the PSI produced too many structures of unknown function our analysis reveals that by using a range of bioinformatics tools and resources we are able to provide functional annotations for more than 90% of the structures solved by the MCSG. Structures of unknown function have helped stimulate the development of methods such as ProFunc that can predict function from structure. ProFunc analysis complements sequence analysis by both adding weight and specific detail to predicted function and by suggesting function where sequence methods have failed to do so.

## Authors' contributions

RL carried out the ProFunc analyses, TdeB carried out the nsSNP analyses, and DL carried out all other analyses and drafted the manuscript. All authors participated in the design of the study, helped draft the manuscript, and read and approved the final manuscript.

## Supplementary Material

Additional file 1 **Additional ProFunc example**. Results and Discussion, Figure, and Reference showing how ProFunc can be used to refine, add detail to, and support a protein function prediction. Click here for file
