# Supplementary material for: 1,000 structures and more from the MCSG
Source: BMC Struct Biol. 2011 Jan 10;11:2. doi: 10.1186/1472-6807-11-2 (PMC3024214; doi:10.1186/1472-6807-11-2)
Supplement: Additional file 1 — Additional ProFunc example. Results and Discussion, Figure, and Reference showing how ProFunc can be used to refine, add detail to, and support a protein function prediction. [file 1472-6807-11-2-S1.DOC]

## **Additional ProFunc example**

**Results and Discussion**

PDB entry 1sfs is an uncharacterized protein from *Geobacillus stearothermophilus* that achieves a ‘possible’ status following sequence analysis. PSI-BLAST suggests lysozyme activity (EC 3.2.1.17) or N-acetylmuramoyl-L-alanine amidase activity (EC 3.5.1.28) while PRC against Pfam suggests glycosyl hydrolases family 25, a family characterized by lysozyme activity. The top reverse template match, rated as certain, is to PDB entry 1jfx, the structure of cellosyl, a 1,4-beta-N-acetylmuramidase M1 (EC 3.2.1.17) from *Streptomyces coelicolor*. This enzyme, which is similar to the lysozymes, cleaves the beta-1,4-glycosidic bond between N-acetylmuramic acid and N-acetylglucosamine units. It also has a beta-1,4-N,6-O-diacetylmuramidase activity [1]. The overall sequence identity between these two proteins is 25.3%. However, the reverse template search returns a number of significant matches between the structures of these proteins, the highest-scoring (residues Trp182-Tyr184-Asn200 in 1sfs matching Trp191-Tyr193-Asn205 in 1jfx) having an E-value of 4.4×10^-8^. The folds of the structures are the same. When the structures are superposed, and the residues in equivalent positions within a 10Å sphere of the template centre are compared, the local sequence identity shoots up to 43.9%. This suggests a fairly high level of local sequence conservation at the site of the template match. And indeed, the catalytic residues in 1jfx are very close to this site. They are Asp9, Asp98 and Glu100, which correspond to Asp9, Asn102 and Glu104 in 1sfs. Figure S1 shows the superposition of the two protein structures, with the known catalytic residues in 1jfx shown as yellow sticks, and the equivalent residues in 1sfs shown as red sticks. The similarities in the structures of these proteins, particularly near the catalytic site of the known muramidase, strongly suggest both an evolutionary relationship between them and a similarity of their biochemical functions, despite their low overall sequence similarity. In this example ProFunc has added weight and specific detail to a possible annotation suggested by sequence analysis.

**Figure**


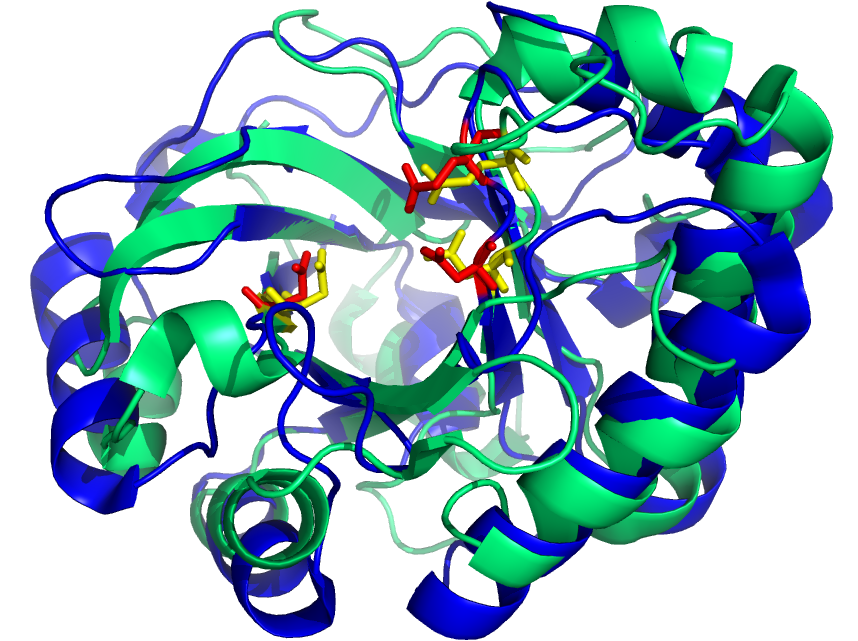


**Figure 1.** **Refining function prediction using ProFunc**. Structural superposition of an uncharacterised protein with a possible functional annotation following sequence analysis (PDB entry 1sfs, in blue) and its top reverse template match, a bacterial muramidase (PDB entry 1jfx, in green). The folds of the two proteins are similar. The residues depicted by yellow sticks are the known catalytic residues in 1jfx (Asp9, Asp98 and Glu100), while the red sticks show the equivalenced residues in 1sfs (Asp9, Asn102 and Glu104).

**Reference**

1. Rau A, Hogg T, Marquardt R, Hilgenfeld R: **A new lysozyme fold. Crystal structure of the muramidase from *Streptomyces coelicolor* at 1.65 A resolution.** *J Biol Chem* 2001, **276**:31994-31999.
